# Disomic Substitution of 3D Chromosome with Its Homoeologue 3E in Tetraploid *Thinopyrum elongatum* Enhances Wheat Seedlings Tolerance to Salt Stress

**DOI:** 10.3390/ijms24021609

**Published:** 2023-01-13

**Authors:** Jian Zeng, Chunli Zhou, Zaimei He, Yi Wang, Lili Xu, Guangdeng Chen, Wei Zhu, Yonghong Zhou, Houyang Kang

**Affiliations:** 1 College of Resources, Sichuan Agricultural University, Chengdu 611130, China; 2 State Key Laboratory of Crop Gene Resource Exploration and Utilization in Southwest China, Sichuan Agricultural University, Chengdu 611130, China; 3 Triticeae Research Institute, Sichuan Agricultural University, Chengdu 611130, China

**Keywords:** tetraploid *Thinopyrum elongtatum*, distant hybridization, physiological response, root adaption, salt tolerance

## Abstract

The halophytic wild relatives within Triticeae might provide valuable sources of salt tolerance for wheat breeding, and attempts to use these sources of tolerance have been made for improving salt tolerance in wheat by distant hybridization. A novel wheat substitution line of K17-1078-3 was developed using common wheat varieties of Chuannong16 (CN16), Zhengmai9023 (ZM9023), and partial amphidiploid Trititrigia8801 (8801) as parents, and identified as the 3E(3D) substitution line. The substitution line was compared with their parents for salt tolerance in hydroponic culture to assess their growth. The results showed that less Na^+^ accumulation and lower Na^+^/K^+^ ratio in both shoots and roots were achieved in K17-1078-3 under salinity compared to its wheat parents. The root growth and development of K17-1078-3 was less responsive to salinity. When exposed to high salt treatment, K17-1078-3 had a higher photosynthesis rate, more efficient water use efficiency, and greater antioxidant capacity and stronger osmotic adjustment ability than its wheat parents. In conclusion, a variety of physiological responses and root system adaptations were involved in enhancing salt tolerance in K17-1078-3, which indicated that chromosome 3E possessed the salt tolerance locus. It is possible to increase substantially the salt tolerance of wheat by the introduction of chromosome 3E into wheat genetic background.

## 1. Introduction

Soil salinization is a growing abiotic stress for agriculture worldwide, which impedes plant growth and decreases grain production worldwide [1]. Approximately 6% of land area is threatened by salinity, including 20% of arable land and 33% of irrigated land [2,3]. More than 50% of agricultural land will be salinized by 2050 due to climate change, land clearing, and unsustainable irrigation, which will pose a serious threat to agricultural sustainability [4]. Soil salinity injures plant growth severely by decreasing growth rate, accelerating leaf senescence, reducing photosynthesis ability, and affecting reproductive development, thus resulting in the yield loss [5]. Under salt stress, excess Na^+^ is absorbed by the roots and transported into the shoots, subsequently disturbing metabolic processes and ion homeostasis [5,6]. The resulting osmotic stress and ion toxicity cause growth inhibition, developmental change, and metabolic disorder [7,8]. In order to cope with salt stress, plants have developed a series of strategies, mainly including osmotic tolerance, Na^+^ exclusion, and tissue tolerance [9]. The osmotic protection as a general response to salt is achieved by the osmolytes accumulation, such as proline, sugar alcohols, and sorbitol in the cytoplasm for osmotic adjustment [10]. While efficient Na^+^ exclusion is often mediated by the plasma membrane SOS1 Na^+^/H^+^ antiporter, avoiding Na^+^ accumulation in cytosol and maintaining cellular osmotic and turgor pressure [11]. In addition, Na^+^ affects photosynthesis by disrupting proton-motive force and chloroplast function as well as interfering with the activity of CO_2_-fixing enzymes [12]. In turn, the good photosynthetic performance can ensure the available resources allocation to osmotic adjustment in response to salt stress. The root is the first tissue directly affected by salt stress as it is exposed to salinity in the rhizosphere [13]. Therefore, the morphological and physiological traits of the root system under salt stress reflect the salt tolerance in plants [14,15]. Furthermore, plants are the most sensitive to salinity at the seedling stage other than flowering and grain filling stage [16]. Consequently, the evaluation of salt tolerance at the seedling stage has always been a concern. The relationship between salinity tolerance and morpho-physiological responses needs to be investigated at the early vegetative stage.

Wheat is the third major cereal crop after corn and rice. The progress in wheat salt tolerance breeding is limited by the low genetic variability of currently available germplasm and the lack of precise indices of physiological and agronomic traits related to salt stress [17]. In wheat, enhancing salt tolerance largely depended on reducing Na^+^ uptake and tissue accumulation [18]. The particularity of historical events in the formation of hexaploid wheat increases physiological and ecological plasticity, which may result in the enhanced tolerance to biotic and abiotic stresses and the wide-ranging adaptability [19]. The wild relatives to wheat contain a large number of gene resources for stress resistance, including some halophytes or salt tolerance genes. By means of distant hybridization, these potential genetic resources are transferred into wheat to further enhance the salt tolerance of wheat. *Thinopyrum elongatum* (syn. *Lophopyrum elongatum*, *Agropyron elongatum*, or *Elytrigia elongata*) is an important promising gene reservoir for improving cereal and forage crops [20,21,22]. In response to salt stress, the introduction of chromosome E from *Th. elongatum* into wheat increases the salt tolerance of wheat due to its greater Na^+^ exclusion and higher K^+^ retention [23,24,25]. In addition, the transfer of genetic materials from *Th. elongatum* into wheat can promote better adaption to some adverse environmental conditions, such as disease infection, cold, and drought [26,27]. Moreover, several sources of improved Na^+^ exclusion are known to reside on different chromosomes in various genomes of wild species related to wheat; further works to illuminate the underlying mechanisms and then to pyramid the controlling genes are pursuing. This provides a good opportunity to broaden the genetic diversity and contribute to extend salt tolerance levels beyond the existing cultivated wheat varieties. However, our understanding of the physiological basis underlying the E chromosomes in conferring salt tolerance to wheat is still limited.

In this study, we have obtained a new substitution line using distant hybridization between wheat and partial amphidiploid derived from tetraploid *Th. elongatum.* An integrated analysis was conducted and aimed (1) to identify the chromosome composition of the substitution line, and (2) to investigate the characteristics in detail for its salt tolerance and growth performance.

## 2. Results

### 2.1. Genome Identification of K17-1078-3

**Figure 1:**
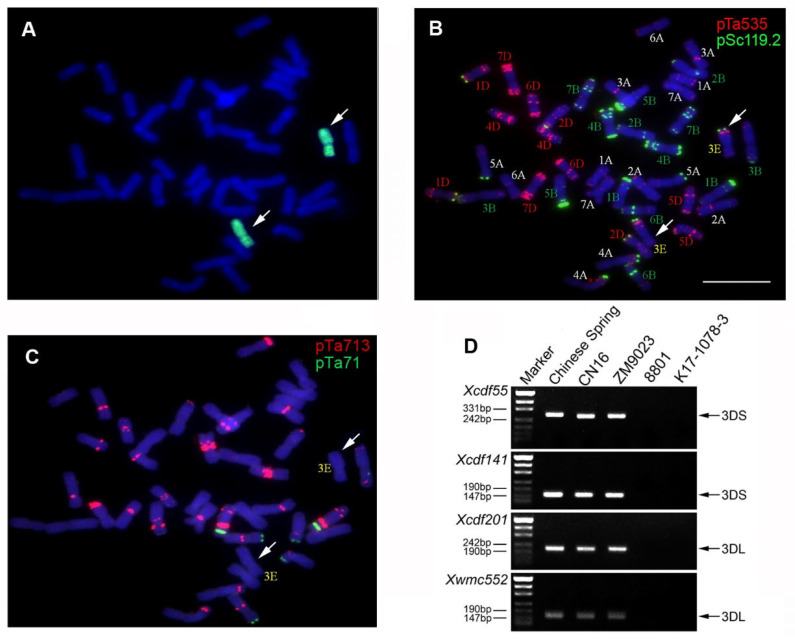
GISH, FISH, and molecular identification of the wheat substitution line K17-1078-3. The probes used for in situ hybridization were genomic DNA of tetraploid *Th. elongatum* (**A**); pSc119.2 and pTa535 (**B**); pTa713 and pTa71 (**C**). The white arrows indicated 3E chromosomes. The black arrows showed the diagnostic amplification products of SSR markers of Xcfd55, Xcfd141, Xcfd201, and Xwmc552 (**D**). Scale bar: 10 µm.

GISH analysis identified K17-1078-3 as having two intact E-genome chromosomes with chromosome number of 2*n* = 6*x* = 42 (Figure 1A). FISH analysis revealed that the red or green fluorescence signal was confirmed at the distal end of the long arm of chromosome 3E (Figure 1B,C). Moreover, there were specific amplicons to chromosome 3D in Chinese spring, CN16, and ZM9023, but not in 8801 and K17-1078-3 using SSR markers analysis, which indicated the absence of chromosome 3D in K17-1078-3 (Figure 1D). Therefore, K17-1078-3 is a novel wheat-tetraploid *Th. elongatum* 3E(3D) substitution line.

### 2.2. Biomass Accumulation in Response to Salinity

**Figure 2:**
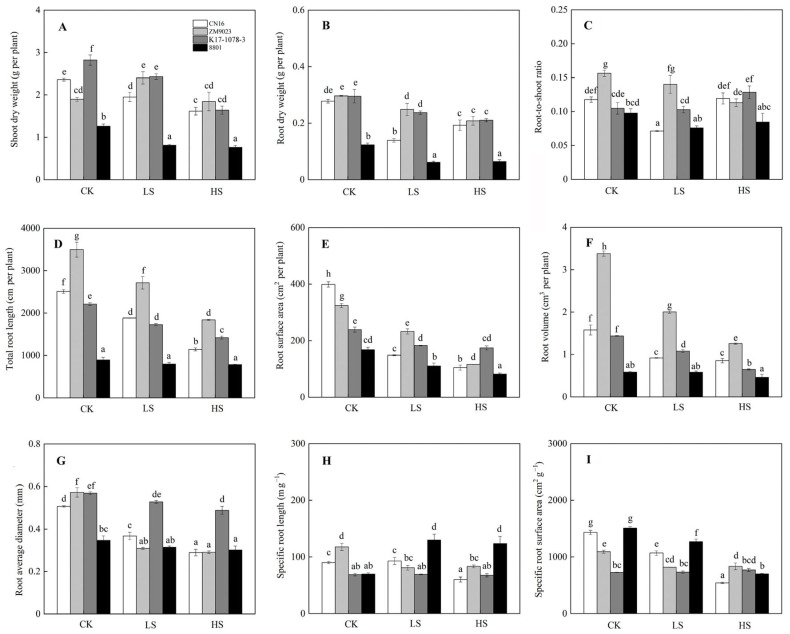
Shoot dry weight (**A**); root dry weight (**B**); root-to-shoot ratio (**C**); total root length (**D**); root surface area (**E**); root volume (**F**); root average diameter (**G**); specific root length (**H**) and specific surface area (**I**) variations in K17-1078-3 and its parents subjected to different salinity levels. Different letters indicated significant difference due to salt treatments according to Duncan’s multiple range tests. CK, LS, and HS referred to NaCl concentrations of 0, 50, and 200 mM.

Salt stress significantly inhibited the plant growth. The SDW and RDW in four wheat genotypes decreased under both LS and HS treatments with the exception of ZM9023. However, there was no significant difference in SDW and RDW of 8801 under LS and HS treatments (Figure 2). Salinity exposure changed the R/S in K17-1078-3, which was significantly higher under HS treatment than under CK and LS treatments. No significant change in R/S was observed in 8801 under salt stress. Meanwhile, the TRL, RSA, RV, and ARD in wheat genotypes decreased when exposed to salinity, with CN16 and ZM9023 showing a significant higher reduction compared to K17-1078-3 and 8801 (Figure 2). In addition, the SRL and SRA in CN16 and ZM9023 decreased to some extent under salt stress. However, the SRL significantly increased in 8801 under LS and HS treatments, and K17-1078-3 had no significant change in SRL and SRA under salt stress.

### 2.3. Photosynthetic Traits in Response to Salinity

**Figure 3:**
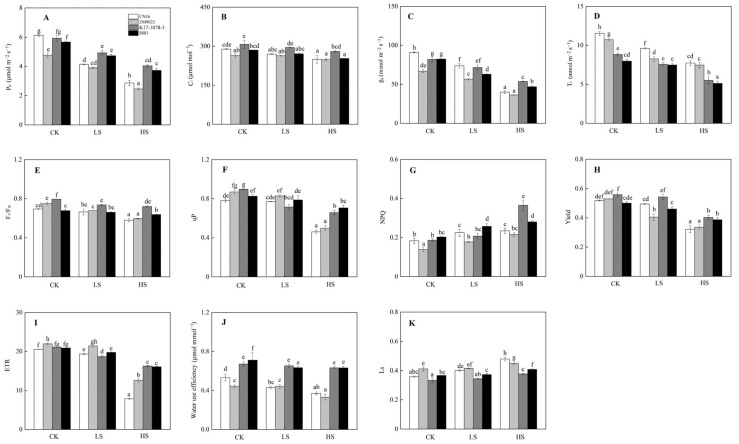
P_n_ (**A**); C_i_ (**B**); g_s_ (**C**); T_r_ (**D**); F_v_/F_m_ (**E**); qP (**F**); NPQ (**G**); yield (**H**); ETR (**I**); water use efficiency (**J**); and L_s_ (**K**) variations in K17-1078-3 and its parents subjected to different salinity levels. Different letters indicated significant difference due to salt treatments according to Duncan’s multiple range tests. CK, LS, and HS referred to NaCl concentrations of 0, 50, and 200 mM.

Salt exposure inhibited photosynthetic ability in four wheat genotypes with K17-1078-3 and 8801 showing higher P_n_ and g_s_, but lower T_r_ than CN16 and ZM9023 under LS and HS treatments (Figure 3). K17-1078-3 and 8801 had higher WUE than CN16 and ZM9023 under all treatments. K17-1078-3 and 8801 had lower Ls than CN16 and ZM9023 under LS and HS treatments (Figure 3). Salt stress changed the characteristics of chlorophyll fluorescence, especially under HS treatment. HS treatment significantly reduced F_v_/F_m_, qP, yield and ETR, and the decreases of CN16 and ZM9023 were greater than those of K17-1078-3 and 8801. K17-1078-3 had significantly higher NPQ than the other three wheat genotypes under HS treatment (Figure 3).

### 2.4. Osmotic Adjustment in Response to Salinity

**Figure 4:**
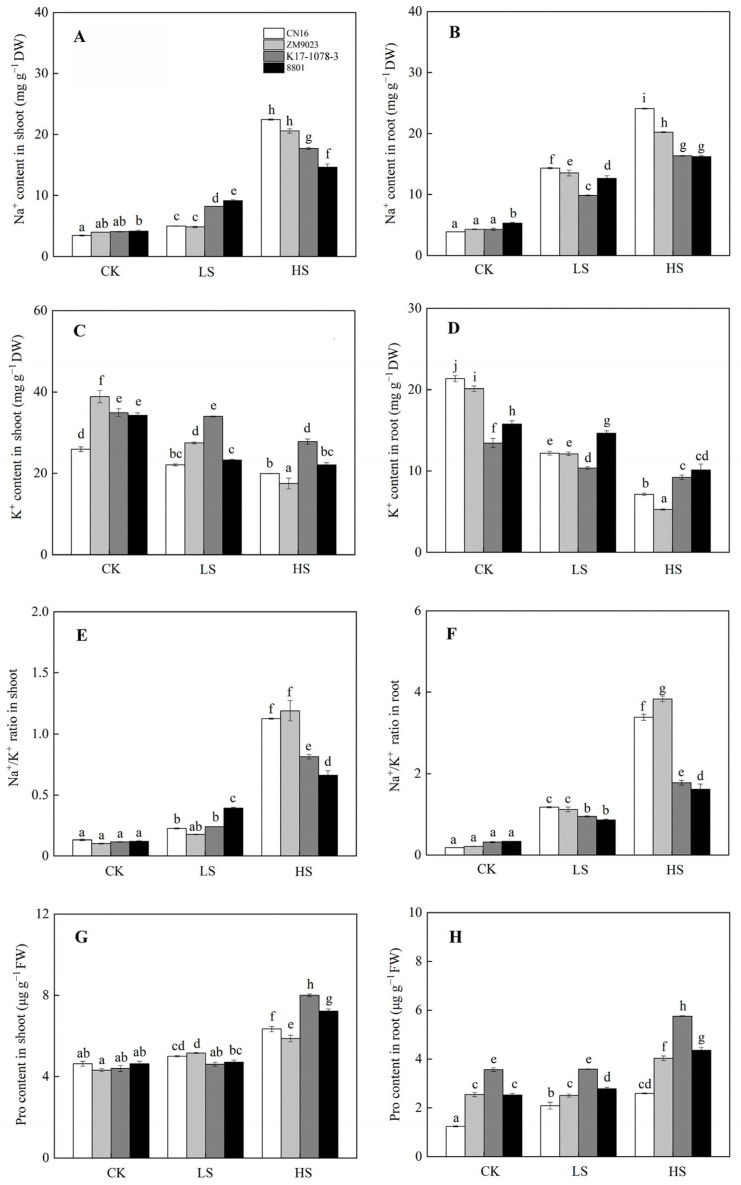
The Na^+^ accumulation in shoot (**A**) and root (**B**); K^+^ accumulation in shoot (**C**) and root (**D**); Na^+^/K^+^ ratio of shoot (**E**) and root (**F**); Pro content in shoot (**G**) and root (**H**) variations of K17-1078-3 and its parents subjected to different salinity levels. Different letters indicated significant difference due to salt treatments according to Duncan’s multiple range tests. CK, LS, and HS referred to NaCl concentrations of 0, 50, and 200 mM.

Salinity significantly increased the Na^+^ content in shoots and roots. CN16 and ZM9023 had higher Na^+^ accumulation in shoots under HS treatment and in roots under LS and HS treatments than K17-1078-3 and 8801 (Figure 4). Meanwhile, K17-1078-3 had significantly higher K^+^ content in shoots than that of the other three wheat genotypes, and maintained a lower decline in K^+^ content of roots compared to CN16 and ZM9023 under LS and HS treatments (Figure 4). Na^+^/K^+^ ratio followed a similar pattern to Na^+^ content in shoots and roots. K17-1078-3 and 8801 had a consistently lower Na^+^/K^+^ ratio in roots under LS and HS treatments and in shoots only under HS treatment than CN16 and ZM9023. Moreover, HS treatment significantly increased the Pro content in shoots and roots compared to CK condition, with K17-1078-3 and 8801 showing higher than CN16 and ZM9023. There was no significant difference in the Pro content of K17-1078-3 and 8801 between CK and LS treatments (Figure 4). Compared with CN16 and ZM9023, K17-1078-3 and 8801 had significantly lower relative conductivity in shoots and roots under HS treatment, and there was no difference in shoots among them under LS treatment. No significant change in relative conductivity was observed in both shoots and roots of K17-1078-3 and 8801 under LS and HS treatments.

### 2.5. Antioxidant Defense in Response to Salinity

**Figure 5:**
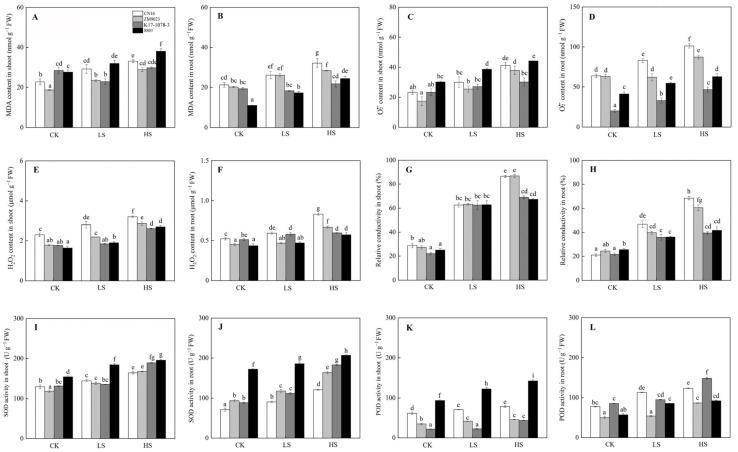
The MDA content in shoot (**A**) and root (**B**); O_2_^•−^ content in shoot (**C**) and root (**D**); H_2_O_2_ content in shoot (**E**) and root (**F**); relative conductivity in shoot (**G**) and root (**H**); SOD activity in shoot (**I**) and root (**J**); and POD activity in shoot (**K**) and root (**L**) variations of K17-1078-3 and its parents subjected to different salinity levels. Different letters indicated significant difference due to salt treatments according to Duncan’s multiple range tests. CK, LS, and HS referred to NaCl concentrations of 0, 50, and 200 mM.

In general, the contents of MDA, H_2_O_2_, and O_2_^•−^ increased because of salt stress, with CN16 and ZM9023 exhibiting higher content than K17-1078-3 and 8801 in roots (Figure 5). Salt stress increased the activities of SOD and POD in both shoots and roots. The SOD activity of 8801 was significantly higher than that of the other three wheat genotypes, and a similar pattern was observed in POD activity of the shoots under salinity (Figure 5). In particular, the SOD activity in both shoots and roots, and the POD activity in roots of K17-1078-3 were considerably higher than those of CN16 and ZM9023 under HS treatment.

## 3. Discussion

There is considerable variability in salt tolerance amongst members of the Triticeae, with the tribe even containing a number of halophytes. These halophytic wild relatives may also provide valuable salt tolerance resources for cereal crops and genetic improvement of wheat salt tolerance. It is of great significance to explore the genetic contribution related to salt tolerance in wild relatives for wheat salt improvement. *Lophopyrum elongatum* (synonym *Th. elongatum*) grows in saline environment around the Mediterranean and survived exposure to 500 mM NaCl, indicating a highly salt-tolerant relative of wheat [28]. Furthermore, a number of resistance genes from *L. elongatum* have been transferred into wheat in the form of amphidiploid, addition, substitution, and translocation lines, which exhibited higher tolerance to salinity than the wheat parent [20]. This may be attributed to greater Na^+^ exclusion and increased K^+^ level [24]. In this study, we have developed a novel wheat substitution line of K17-1078-3. Integrative analyses of GISH, FISH, and SSR specific markers confirmed the substitution of chromosome 3D with chromosome 3E in K17-1078-3 with 42 chromosomes.

### 3.1. Effect of Chromosome 3E on Root Growth and Photosynthesis

At plant organ level, the aboveground organs are more sensitive to salinity than the belowground organs [5]. The available evidence on the effect of salinity on the root is mainly confined to the root mass. The root mass reduction and root morphological trait modification caused by salt stress in wheat is evident and root surface area at a specific phytomer position is closely relation to salt tolerance [29]. In this study, salinity significantly inhibited root growth as manifested by the obvious reduction in TRL, RSA, ARD, and RV. The effect of salt stress on root growth inhibition in CN16 and ZM9023 was greater than in K17-1078-3 and 8801, but the difference between K17-1078-3 and 8801 was not significant. A better root growth maintenance during salt exposure was recorded in K17-1078-3 and 8801, which suggested that the presence of chromosome 3E contributed to root growth. Good root growth and development under salt stress was beneficial for the uptake of nutrients and water in order to facilitate the utilization of substrates and metabolic energy [29]. Therefore, the salt tolerance of K17-1078-3 may be achieved by substituting chromosome 3D with chromosome 3E to maintain root growth. In addition, a high growth rate of plants in the absence of salinity is a better measure of productivity in saline condition than physiological tolerance of salinity at the early growth stage [30]. Our findings also showed that K17-1078-3 and 8801 possessed greater relative growth rate and biomass accumulation than CN16 and ZM9023 under CK condition, indicating that the presence of chromosome 3E was beneficial to maintain the productivity, thereby implying the potential tolerance to salt stress. Consequently, it was reasonable that the 3E(3D) substitution line of K17-1078-3 was capable of a stronger tolerance to salinity, which uncovered the genetic contribution of substituting chromosome 3D with chromosome 3E to the salt tolerance in wheat.

In response to salt stress, photosynthesis directly affects the available resources for plant growth. Under salt stress, Na^+^ mediated the balance destruction between pH and electron potential change results in the reduction of CO_2_-fixing enzymes activities and energy production, which, therefore, affects the photosynthetic rate [12]. We found a greater photosynthetic ability, as evidenced by higher P_n_, g_s_, T_r_, F_v_/F_m_, qP, yield, and ETR, in K17-1078-3 and 8801 exposed to salinity, especially under HS treatment. In addition, the Ls values of K17-1078-3 and 8801 under salt stress were lower than those of CN16 and ZM9023. These results indicated that chromosome 3E contributed to the photosynthesis improvement in wheat, thus providing more assignable resources to cope with salt stress. Plants cannot effectively absorb light energy under adverse conditions, excessive light energy tends to cause photo-inhibition and photosynthesis decline. Photo-inhibition is avoided, in part, by NPQ activation, which can dump a large faction of excitation energy, thereby protecting photosynthetic apparatuses [31]. In this study, K17-1078-3 and 8801 had significantly higher NPQ than CN16 and ZM9023, while NPQ was significantly increased in K17-1078-3 compared with 8801 under HS treatment. This indicated that K17-1078-3 had a stronger ability to protect photosynthetic apparatuses in response to salt stress. In the same way, salt stress resulted in the reduction of water use efficiency. K17-1078-3 and 8801 exhibited a significantly higher water use efficiency compared with CN16 and ZM9023, suggesting that chromosome 3E contributed to reducing water loss and improving salt adaptability or tolerance in wheat.

### 3.2. Effect of Chromosome 3E on Na^+^ Accumulation and K^+^ Acquisition

The most essential traits are excluding Na^+^ and Cl^−^ and depending on organic solutes for osmotic adjustment in plants under salt stress [32]. The distal end of the long arm of homoeologous 3A or 3D replaced with diploid *L. elongatum* chromatin controls Na^+^ exclusion, which contributes to improving salt tolerance in wheat [26]. In wheat, excluding Na^+^ from shoots and tolerating a high internal level of Na^+^ are essential strategies in response to salt stress [18]. Salt tolerance and Na^+^ accumulation among wheat genotypes are independent of each other, and the salt-tolerant genotype exhibits a higher Na^+^/K^+^ ratio than the salt-sensitive genotype [33,34]. In addition, root system strategies in response to salinity are partially correlated with the Na^+^/K^+^ ratio in shoots [35]. In this study, K17-1078-3 and 8801 exhibited a lower Na^+^/K^+^ ratio in shoots under HS treatment and in roots under LS and HS than CN16 and ZM9023 (Figure 4), indicating that the chromosome 3E was able to minimize Na^+^ accumulation in the plant. On the other hand, K17-1078-3 and 8801 showed a lower K^+^ content in roots than CN16 and ZM9023 under CK condition, and the lower reduction in K^+^ content caused by salinity was available in K17-1078-3 and 8801. This suggested that chromosome 3E played an important role in the low requirement and good homeostasis maintenance for K^+^ to support plant growth exposed to salinity. Therefore, the newly developed substitution lines of K17-1078-3 will promote the salt improvement in wheat.

### 3.3. Effect of Chromosome 3E on Antioxidant Defense

Excess Na^+^ accumulation is toxic to many enzymes in the cell, affecting physiological and metabolic processes. The accumulation of organic osmolytes, such as proline, soluble protein, and soluble sugar, under salt stress protects the cell by balancing the osmotic strength of the cytosol with that of the vacuole and external environment [36]. In addition to their role as cytosolic osmolytes, these solutes also stabilize the structure and function of biological macromolecules [37]. The higher proline content in the shoots of K17-1078-3 and 8801 was observed under HS treatment, especially for roots under both LS and HS treatments, which helped to increase antioxidant enzymes activities and decrease electrolyte leakage (Figure 5). Proline is known to act as a reactive oxygen species (ROS) scavenger, redox buffer, or molecular chaperone, stabilizing proteins and membrane structures under stress conditions [38]. Excess ROS can damage plant tissue by perturbing enzyme activity, the cell wall, and membrane function during salinity stress. In this study, salinity caused the increases in H_2_O_2_, O_2_^•−^, and MDA, but K17-1078-3 and 8801 had significantly lower values in roots compared to CN16 and ZM9023 under LS and HS treatments (Figure 5). This indicated that K17-1078-3 and 8801 suffered from lower oxidative damage to roots under salt stress, possessing the salt tolerance potential in the presence of chromosome 3E. On the other hand, plants are equipped with antioxidant defense systems in the form of enzymatic components, such as SOD, POD, APX, and CAT, to counter the overproduction of ROS and avoid the oxidative damage. The antioxidant enzyme activity was positively correlated to salinity tolerance in plants [39,40]. In this study, the antioxidant enzymes activities of both shoots and roots increased as a response to salt stress, and we found the SOD activity of shoots and roots and the POD activity of roots in K17-1078-3 were significantly higher than that of CN16 and ZM9023 under HS treatment. The high SOD and POD activities of shoots were particularly prominent in 8801 under all treatments. These results allowed us to conclude that the increase of antioxidant enzymes activities in K17-1078-3 was involved in ROS detoxification in response to salt stress and highlighted the genetic contribution of chromosome 3E to salt tolerance improvement in wheat.

## 4. Materials and Methods

### 4.1. Substitution Line Production

Two wheat cultivars, Chuannong16 (CN16, 2*n* = 6*x* = 42, genome AABBDD) and Zhengmai9023 (ZM9023, 2*n* = 6*x* = 42, genome AABBDD), and a partial amphidiploid Trititrigia8801 (8801, 2*n* = 6*x* = 42, genome AABBEE) were used to produce the novel substitution line. Then, 8801 was generated by hybridization between *Triticum durum* (2*n* = 4*x* = 28, genome AABB) and tetraploid *Th. elongatum* (2*n* = 4*x* = 28, genome EEEE). We firstly crossed 8801 with CN16, and thereafter the F_1_ hybrids were crossed with ZM9023, as the male parent, to obtain the BC_1_F_1_ generation. A substitution line designated as K17-1078-3 was developed through three consecutive cycles of self-pollination.

### 4.2. Genome Identification

Actively growing root tips of K17-1078-3 were collected from germinating seeds and treated with nitrous oxide for 2 h and then immersed in 90% acetic acid for 10 min. Slides were prepared for genomic in situ hybridization (GISH) as previously described by Han et al. [41]. Tetraploid *Th. elongatum* genomic DNAs were labeled with fluorescein-12-dUTP using nick translation mix (Thermo Fisher Scientific, Eugene, OR, USA). Sheared genomic DNAs of CN16 and ZM9023 were used as blocking DNA. The GISH procedure and signal detection were conducted following the method of Han et al. [41]. Photomicrographs of GISH chromosomes were taken with an Olympus BX-51 microscope (Olympus, Tokyo, Japan) equipped with CCD camera. Fluorescence in situ hybridization (FISH) was performed with oligonucleotide probes of Oligo-pSc119.2, Oligo-pTa535, Oligo-pTa71, and Oligo-pTa713 with a specific one for chromosome 3E [27]. Probe labeling, hybridization, and signal detection were identical to the GISH procedure. In addition, four simple sequence repeats markers (Xcfd55, Xcfd141, Xcfd201, and Xwmc552) distributed on chromosome 3DS and 3DL in wheat were used to determine chromosome 3D chromatin in K17-1078-3 (Table 1). The PCR mixture and amplification procedure were conducted as described by Somers et al. [42] with minor modifications.

### 4.3. Growth Conditions and Salt Treatments

Salt tolerance was evaluated using a hydroponic system. Uniformly sized seeds of K17-1078-3 and its parents were sterilized in 3% sodium hypochlorite for 5 min, followed by three lots of rinsing with deionized water, and then germinated on moist filter paper in Petri dishes at room temperature for 3 days. At the three leaves stage, the seedlings were transplanted into plastic pots (three seedlings per pot) with Hoagland’s solution supply. Three levels of salinity stress were used to compare the tolerance to salinity: no applied NaCl (control, CK); 50 mM NaCl (low salinity, LS); and 200 mM NaCl (high salinity, HS). The pH of solutions was maintained within a range of 5.5–6.5. The pots were placed in a randomized block design in a growth chamber with a 16 h/8 h light/dark cycle, a light intensity of 300 µmol m^−2^ s^−1^, 22/14 °C day/night, and 70% relative humidity. The nutrient solution was renewed every 3 days.

### 4.4. Root Morphology and Photosynthesis Measurements

After 7 days of growth, the seedlings were harvested for further analysis. The roots were washed and immediately scanned at 300 dpi and analyzed with the Win-RHIZO software (Regent Instruments Inc., Sainte Foy, QC, Canada) to obtain total root length (TRL), average root diameter (ARD), root volume (RV), and root surface area (RSA). The specific root length (SRL) and specific root surface area (SRA) were calculated as the ratio of TRL and RSA to RDW, respectively. Subsequently, shoot dry weight (SDW) and root dry weight (RDW) were determined after drying to a constant weight at 80 °C. The root-to-shoot ratio (R/S) was calculated as the ratio of RDW/SDW. For determination of total N and P contents, the dried tissue samples were weighed, ground into a powder, and digested in a mixture of H_2_SO_4_ and H_2_O_2_ before analysis by the Kjeldahl method and vanadium molybdate blue colorimetric method, respectively. The K^+^ and Na^+^ contents were estimated using a flame photometer (AP1500, Aopu Analytical Instruments Inc., Shanghai, China) and the analyses was verified using a certified plant tissue standard (GBW07604, Institute of Geophysical and Geochemical Exploration, Chinese Academy of Geological Sciences).

Gas exchange measurements were conducted for the uppermost, fully expanded leaves in three randomly chosen individuals from each treatment between 08:00 and 11:30 am using Li-6400 portable photosynthesis system (LI-COR Inc., Lincoln, NE, USA). In the assimilation chamber, external CO_2_ concentration was set to 360 ± 5 µmol mol^−1^, 70–75% relative humidity, and light intensity was constant at 300 µmol photons m^−2^ s^−1^. The following photosynthetic parameters were recorded for three plants per replicate: net photosynthetic rate (P_n_), intercellular CO_2_ concentration (C_i_), stomatal conductance (g_s_), and transpiration rate (T_r_) when stable photosynthetic rates were achieved. Water use efficiency (WUE) was calculated as P_n_ to T_r_ ratio and stomatal limitation value (L_s_) as ambient CO_2_ concentration to intercellular CO_2_ concentration ratio. Chlorophyll fluorescence was measured on the leaves used for photosynthesis determination using the MINI version of Imaging-PAM (Heinz Walz GmbH, Effeltrich, Germany). After plants were dark-adapted for 30 min, the leaves were exposed to a weak modulated measuring beam and a saturation pulse to obtain the minimum fluorescence (F_o_) and the maximum fluorescence (F_m_). The maximum photosystem II quantum efficiency (F_v_/F_m_) was calculated as the formula of F_v_/F_m_ = (F_m_ − F_o_)/F_m_. The actinic photo irradiance of 531 µmol m^−2^ s^−1^ for 240 s was applied to obtain the steady-state fluorescence yield (F_s_); subsequently, a saturating blue light pulse (2400 µmol m^−2^ s^−1^, 0.8 s) was applied to achieve the light-adapted maximum fluorescence (F_m_’). The light-adapted initial fluorescence (F_o_’) was estimated according to Oxborough and Barker [43]. The energy for the dissipative processes was calculated as follows: effective photochemical quantum yield of photosystem II (Yield) = (F_m_’ − F_s_)/F_m_’; non-photochemical quenching (NPQ) = (F_m_ − F_m_’)/F_m_’; photochemical quenching (qP) = (F_m_’ − F_s_)/(F_m_’ − F_o_’). The apparent electron transport rate (ETR) was estimated with the equation, ETR = Yield × PPFD × 0.5 × 0.84, where PPFD is photosynthetic photon flux density incident on the leaf, the factor 0.5 is used on the assumption of an equal distribution of energy between the two photosystems, and the factor 0.84 represents an assumed leaf absorbance.

### 4.5. Antioxidant Defense Assays

The superoxide dismutase (SOD) activity was determined by measuring its ability to inhibit the photochemical reduction of nitroblue tetrazolium (NBT) as described by Beauchamp and Fridovich [44]. The peroxidase (POD) activity was determined by the method of Takahama and Egashira [45]. O_2_^•−^ and H_2_O_2_ production levels were determined following the description of Wang and Luo [46] and Bernt and Bergmeyer [47], respectively. Malondialdehyde (MDA) content was measured according to the method of Dhindsa et al. [48], and proline (Pro) content was measured as described by Singh et al. [49]. In addition, the relative conductivity was estimated using an electrical conductivity meter using the method of Lutts et al. [50].

### 4.6. Statistical Analysis

All experimental data were calculated as the mean ± standard deviation of three replicates. The differences between treatments were analyzed using a statistical software of SPSS 27.0 (SPSS Inc., Chicago, IL, USA). In statistical analyses, *p*-values of less than 0.05 were considered to be statistically significant according to Duncan’s multiple range tests.

## Figures and Tables

**Table 1:** SSR markers sequences for chromosome arm location of 3DS and 3DL in wheat were used in this study. F indicated forward primer; R indicated reverse primer.

Marker	Primer Sequences (5′-3′)	Annealing Temperature (°C)	Chromosomal Arm Location	Amplification Size (bp)
Xcfd55	F: CCAGTAGCCGGCCCTACTAT R: GCACGAGATACGGACAATCA	57	3DS	269
Xcfd141	F: CGTAAAGATCCGAGAGGGTG R: TCCGAGGTGCTACCTACCAG	58	3DS	152
Xcfd201	F: ACAAGACCACACCTCCAAGG R: CGGTTTGGGTTTTGTGATCT	55	3DL	206
Xwmc552	F: ACTAAGGAGTGTGAGGGCTGTG R: CTCTCGCGCTATAAAAGAAGGA	58	3DL	149

## Data Availability

All data, models, and code generated or used during the study appear in the submitted article.
